# AMS Reviewer Summary 2024

**DOI:** 10.1002/ams2.70043

**Published:** 2025-02-24

The publication of invaluable papers in the Acute Medicine & Surgery depends on the prompt, careful review of submitted manuscripts. We would like to thank the Editorial Board members and the following experts for reviewing manuscripts from January 1, 2024 to December 31, 2024.

Abe, Ryuzo

Abe, Yoshinobu

Akahoshi, Tomohiko

Akutsu, Hiroyoshi

Amagasa, Shunsuke

Amemiya, Yu

Aokage, Toshiyuki

Aoki, Makoto

Arimoto, Hideki

Ariyoshi, Koichi

Atagi, Kazuaki

Atsumi, Takahiro

Azuma, Kazunari

Chiba, Takuyo

Cho, Kosai

Doi, Kent

Doi, Tomoaki

Ebihara, Takayuki

Endo, Tomoyuki

Fernandez, Luis G.

Fujii, Tomoko

Fujiogi, Michimasa

Fujishima, Seitaro

Fujita, Motoki

Fujita, Satoshi

Fujizuka, Kenji

Fukushima, Hidetada

Funabiki, Tomohiro

Funakoshi, Hiraku

Furukawa, Makoto

Goto, Tadahiro

Goto, Yoshikazu

Goto, Yukari

Hanada, Hiroyuki

Hanajima, Tasuku

Hanaki, Nao

Hara, Yoshitaka

Harada, Masahiro

Hashiguchi, Naoyuki

Hatakeyama, Junji

Hattori, Noriyuki

Hayakawa, Katsura

Hayakawa, Koichi

Hayakawa, Mineji

Hayashi, Hiroyuki

Hayashida, Kei

Hibino, Seikei

Hifumi, Toru

Hiraide, Atsushi

Hirayama, Takahiro

Hirose, Tomoya

Homma, Hiroshi

Homma, Yosuke

Hongo, Takashi

Hosomi, Sanae

Ikeda, Hisato

Inamasu, Joji

Inokuchi, Koichi

Inoue, Shigeaki

Inoue, Takehiro

Inoue, Yoshiaki

Irahara, Takayuki

Ishida, Kenichiro

Ishihara, Satoshi

Ishihara, Tadashi

Isokawa, Shutaro

Iwashita, Yoshiaki

Izawa, Yoshimitsu

Jones, Daryl

Kajino, Kentaro

Kaneko, Tadashi

Kashiura, Masahiro

Katayama, Yusuke

Kikutani, Kazuya

Kiriu, Nobuaki

Kitamura, Tetsuhisa

Kiyota, Kazuya

Koami, Hiroyuki

Kobata, Hitoshi

Kohara, Saeko

Kotani, Joji

Kuboyama, Kazutoshi

Kudo, Daisuke

Kurihara, Tomohiro

Kuroda, Yasuhiro

Kurozumi, Taketo

Kuwana, Tsukasa

Matsui, Satoshi

Matsumura, Yosuke

Matsunaga, Hiroki

Matsushima, Asako

Matsuura, Hiroshi

Matsuyama, Tasuku

Mayumi, Toshihiko

Mitate, Eiji

Mitsunaga, Toshiya

Miyamoto, Kyohei

Mizobata, Yasumitsu

Mizushima, Yasuaki

Mori, Takaaki

Morishita, Koji

Morita, Masanori

Muronoi, Tomohiro

Muroya, Takashi

Naito, Hiromichi

Nakada, Taka‐Aki

Nakae, Hajime

Nakagawa, Yuko

Nakamori, Yasushi

Nakao, Shunichiro

Nishida, Takeshi

Nishimura, Takeshi

Nishimura, Tetsuro

Nishiuchi, Tatsuya

Nishiyama, Kei

Nojima, Tsuyoshi

Nomura, Osamu

Ochiai, Hidenobu

Ogura, Takayuki

Ohshimo, Shinichiro

Ohta, Bon

Oka, Kazuyuki

Okada, Ichiro

Okada, Motoi

Okada, Yohei

Omura, Takeshi

Ono, Yuko

Onodera, Makoto

Oshima, Taku

Osuga, Keigo

Osuka, Akinori

Ota, Koshi

Otani, Norio

Ozaki, Masayuki

Saito, Fukuki

Sakamoto, Ayaka

Sakamoto, Taigo

Sakamoto, Yuichiro

Sakiyama, Yoshio

Sakurai, Atsushi

Sasaki, Junichi

Sasaki, Nobuo

Sassoè, Marco

Sato, Nobuhiro

Sato, Tetsuya

Sato, Yukio

Sekine, Kazuhiko

Shiga, Takashi

Shigemori, Yutaka

Shime, Nobuaki

Shimizu, Keiki

Shimizu, Kentaro

Shinozaki, Koichiro

Shiomi, Naoto

Subbe, Christian

Suehiro, Eiichi

Sueyoshi, Koichiro

Suzuki, Keisuke

Tachino, Jotaro

Tagami, Takashi

Takahashi, Gaku

Takahashi, Nozomi

Takamatsu, Jumpei

Takeyama, Naoshi

Tampo, Akihito

Tanaka, Chie

Tanaka, Hideharu

Tanaka, Yoshihiro

Tanikawa, Atsushi

Tarui, Takehiko

Tatsuishi, Wataru

Terauchi, Yasuo

Tobe, Masaru

Tohira, Hideo

Toida, Chiaki

Tomioka, Joji

Tsunetoh, Satoshi

Tsuruta, Ryosuke

Uchida, Kenichiro

Uemura, Shuji

Umemura, Yutaka

Uno, Shunsuke

Uzura, Masahiko

Varatharajan, Sakthivadivel

Wada, Takeshi

Watanabe, Hiroaki

Yakushiji, Hiromasa

Yamada, Noriaki

Yamahata, Yoshihiro

Yamakawa, Kazuma

Yamamoto, Ryo

Yamashita, Norio

Yasuda, Hideto

Yoshimura, Yuya

Yumoto, Tetsuya

